# P53-MDM2 Pathway: Evidences for A New Targeted Therapeutic Approach in B-Acute Lymphoblastic Leukemia

**DOI:** 10.3389/fphar.2016.00491

**Published:** 2016-12-16

**Authors:** Stefania Trino, Luciana De Luca, Ilaria Laurenzana, Antonella Caivano, Luigi Del Vecchio, Giovanni Martinelli, Pellegrino Musto

**Affiliations:** ^1^Laboratory of Pre-Clinical and Translational Research, IRCCS – Referral Cancer Center of BasilicataRionero in Vulture (PZ), Italy; ^2^CEINGE – Biotecnologie Avanzate S.C.a R.L.Naples, Italy; ^3^Department of Molecular Medicine and Medical Biotechnologies, Universita’ degli Studi di Napoli Federico IINaples, Italy; ^4^Department of Experimental, Diagnostic and Specialty Medicine, Institute of Hematology “L. and A. Seràgnoli,” University of BolognaBologna, Italy; ^5^Scientific Direction, IRCCS – Referral Cancer Center of BasilicataRionero in Vulture (PZ), Italy

**Keywords:** acute lymphoblastic leukemia, p53, MDM2, Nutlin-3a, target therapy

## Abstract

The tumor suppressor p53 is a canonical regulator of different biological functions, like apoptosis, cell cycle arrest, DNA repair, and genomic stability. This gene is frequently altered in human tumors generally by point mutations or deletions. Conversely, in acute lymphoblastic leukemia (ALL) genomic alterations of *TP53* are rather uncommon, and prevalently occur in patients at relapse or with poor prognosis. On the other hand, p53 pathway is often compromised by the inactivation of its regulatory proteins, as MDM2 and ARF. MDM2 inhibitor molecules are able to antagonize p53-MDM2 interaction allowing p53 to exert tumor suppressor transcriptional regulation and to induce apoptotic pathways. Recent preclinical and clinical studies propose that MDM2 targeted therapy represents a promising anticancer strategy restoring p53 dependent mechanisms in ALL disease. Here, we discussed the use of new small molecule targeting p53 pathways as a promising drug target therapy in ALL.

## Introduction

*TP53* is a tumor suppressor gene, located on chromosome 17p13.1, with the main function to prevent cancer transformation (Brady and Attardi, 2010). P53 is a transcription factor that activates or represses a series of target genes exerting different biological functions (Shi and Gu, 2012; Leenders and Tuszynski, 2013). Consequently to a plethora of multiple stress signals, p53 determines cell fate activating apoptosis or maintaining cells at the G1/S regulation point in a reversible cell cycle arrest process; furthermore, it can induce cellular senescence characterized by an irreversible loss of proliferative potential (Demidenko et al., 2010; Timofeev et al., 2013; Burgess et al., 2016). P53 dysfunction can promote the initiation or progression of different human tumors and confer malignant characteristics, such as altered cellular differentiation, genetic instability, and increased metastatic potential (Muller and Vousden, 2013; Bieging et al., 2014). Generally, *TP53* is inactivated in the majority of human solid tumors by missense mutations and deletions impairing transcriptional function of the protein (Olivier et al., 2010; Naccarati et al., 2012; Gibbons et al., 2014). Conversely, in hematological malignancies, where p53 mutations are less recurrent, its activity may be likewise compromised by the alterations of MDM2 (**Table 1**) and ARF (Richmond et al., 2015; Kojima et al., 2016), two regulators of p53. MDM2 (mouse double minute-2) binds p53 regulating its stability and cellular localization. This interaction inhibits p53 mediated transcriptional activity and induces p53 proteasomal degradation (Eischen and Lozano, 2009; Van Maerken et al., 2014). ARF (alternative reading frame), instead, is a tumor suppressor encoded by *CDKN2A* gene, that participates to the regulation of p53, by interacting with MDM2. This binding blocks MDM2 shuttling between the nucleus and cytoplasm avoiding p53 degradation (Maggi et al., 2014; Vivo et al., 2015).

**Table 1:** MDM2 deregulations in various hematological malignancies.

Hematological malignancy	MDM2 deregulation	References
ALL	overexpression	Zhou et al., 1995, 2000; Gu et al., 2008; Zhu et al., 2008
AML	overexpression	Faderl et al., 2000; Kojima et al., 2005; Reis et al., 2016
CLL	overexpression	Haidar et al., 1997; Isin et al., 2012
CML		Trotta et al., 2003; Carter et al., 2015
HL	amplification	Kupper et al., 2001
NHL	overexpression	Pagnano et al., 2001
MCL	amplification, overexpression	Solenthaler et al., 2002; Hernandez et al., 2005
BL	overexpression	Wilda et al., 2004
BCL	overexpression	Riley et al., 2016
DLBCL	overexpression	Davies et al., 2005
MM	overexpression	Teoh et al., 1997; Kryukov et al., 2013; Teoh et al., 2014

*ALL, acute lymphoblastic leukemia; AML, acute myeloid leukemia; CLL, chronic lymphocytic leukemia; CML, chronic myeloid leukemia; HL, Hodgkin’s lymphoma; NHL, non-Hodgkin’s lymphoma; MCL, mantle cell lymphoma; BL, Burkitt’s lymphoma; BCL, B-cell lymphoma; DLBCL, diffuse large B-cell lymphoma; MM, multiple myeloma.*

In acute lymphoblastic leukemia (ALL) MDM2 is overexpressed (Zhou et al., 1995, 2000; Gu et al., 2008) and *CDKN2A* gene is frequently deleted (Usvasalo et al., 2008; Iacobucci et al., 2011).

In this review, we summarized the current knowledge about p53-MDM2 axis in ALL focusing our attention on a new potential therapeutic agent restoring p53 dependent mechanisms in this hematological disease.

## P53 Abnormalities in Acute Lymphoblastic Leukemia

*TP53* mutations were considered infrequent in ALL (Hof et al., 2011; Chiaretti et al., 2013; Saha et al., 2013) and were correlated with cytogenetic alterations, like low hypodiploidy, or MYC-rearrangements (Holmfeldt et al., 2013; Stengel et al., 2014). Moreover, the disruption of both *TP53* alleles was associated with adverse prognosis (Stengel et al., 2014). Also the aberrant methylation could contribute to *TP53* gene inactivation; in particular, Agirre et al. (2003) showed that *TP53* promoter resulted methylated in 8 of out 25 ALL patients and its expression was decreased in all the methylated samples. Other literature data found 13 genes, involved in the *TP53* dependent pathway, down-regulated by hypermethylation in a large cohort of ALL patients at diagnosis. Methylation of at least 1 of the 13 genes was observed in 78% of the patients, which significantly correlated with a higher relapse and mortality rate predicting the clinical outcome of patients (Vilas-Zornoza et al., 2011).

On the other hand, also deregulation of microRNAs was found to be correlated with p53 alteration. In particular, Nucera et al. (2016) focused their attention of *miRNA-126*, a regulator of hematopoietic stem cell quiescence. They found that *mir-126* was highly expressed in human B-ALL and target p53 response genes orchestrating an oncogenic program by down-regulation of p53-dependent pathway. Another microRNA found to have a role as onco-miRNA in ALL was *mir-181a* that down-regulated the expression of tumor suppressor gene *EGR1* (Verduci et al., 2015).

Finally, p53 was also inactivated by the frequent deletion of *CDNK2A* (Usvasalo et al., 2008; Iacobucci et al., 2011) and the overexpression of *MDM2* in ALL patients (Zhou et al., 1995, 2000; Gu et al., 2008).

## Current Treatments of All

B-ALL is a heterogeneous disease on biological and clinical point of view, affecting pediatric, adolescent, adult, and older patients. It prevalently occurs, however, in childhood, in whom the prognosis is more favorable respect to adult patients, reaching a cure rate of 80–90% thanks to multi-agent and intensive combination chemotherapy regimens that have significantly improved the outcome in the pediatric setting (Hunger and Mullighan, 2015; Pui et al., 2015), as well as in that of adolescent and younger adults (Curran and Stock, 2015). In other patients, instead, “conventional” treatments remain unsatisfactory (Marks, 2015; Al Ustwani et al., 2016; Fedorov et al., 2016), due to pharmacologic resistance (Ronson et al., 2016; Seiter, 2016) or toxicity events, above all when aggressive “pediatric-like” protocols are applied (Dias et al., 2016).

A subset of B-ALL shows *t*(9:22) translocation that generates “Philadelphia” chromosome (Ph) encoding a specific BCR-ABL1 tyrosine kinase fusion protein. This alteration occurs in 3–4% of pediatric ALL and about 25% of adult patients, increasing with age: these patients strongly benefit of the BCR–ABL1 tyrosine kinase inhibitors (TKI) as first-line treatment (Malagola et al., 2016). However, although TKI monotherapy induces complete remission rates of 90–100% with low toxicity profile even in older patients (Vignetti et al., 2007; Foa et al., 2011), the combination of TKI with standard chemotherapy is generally required to obtain higher long-term disease free survival in both adults (Fielding et al., 2014; Fielding, 2015) and children (Biondi et al., 2012; Bleckmann and Schrappe, 2016) with Ph positive ALL.

More recently, new therapies seem to be appealing for treatment of refractory/relapsed patients. They are based on monoclonal antibodies targeting antigens, including CD19, CD20, CD22, and CD52, expressed on leukemic blast cell surface (Jabbour et al., 2015). Rituximab, an anti-CD20 antibody, in combination with conventional chemotherapy, has been shown to improve survival in newly diagnosed CD20+ ALL (Maury et al., 2016). Blinatumomab, a T-cell engaging bi-specific single-chain antibody (BiTE) direct to CD19 and CD3, is used as monotherapy in relapsed and refractory ALL, prolonging relapse free survival (Benjamin and Stein, 2016; Le Jeune and Thomas, 2016). Inotuzumab ozogamicin, an anti-CD22 antibody conjugated with a toxin, alone and in combination with chemotherapy, has been promising in relapsed and refractory B ALL (Yilmaz et al., 2015). Several newer monoclonal antibodies (ofatumumab, obinutuzumab, epratuzumab, denintuzumab mafodotin and, moxetumomab pasudotox) are currently under investigation as single agents or in combination with a chemotherapeutic back bone (Farhadfar and Litzow, 2016).

Other novel clinical approaches are related to immunotherapy by engineering of T-cells, derived from patients or allogeneic donors, with synthetic chimeric antigen receptors (CAR T-cells) that activate T cells enhancing their function (Maude et al., 2015; Sadelain et al., 2015).

## Pre-Clinical Evidences of MDM2 Inhibition as a Therapeutic Strategy in Acute Lymphoblastic Leukemia

To improve the outcome of B-ALL patients, novel therapeutic strategies have been developed, like the reactivation of apoptotic pathway by inhibiting MDM2 protein.

Zhang et al. (2014) demonstrated that Nilotinib, a second generation TKI inhibitor, inhibited MDM2 in both Ph^+^ and Ph^-^ ALL cell lines with high MDM2 expression. This inhibition activated a p53-independent apoptosis by down-regulation of the anti-apoptotic protein XIAP. Gu et al. (2008) instead showed a cytotoxic activity of Nutlin-3a, a cis-imidazoline small molecules antagonizing Mdm2-p53 binding, in pediatric ALL with p53 wild-type and over-expressing MDM2. Moreover, they also found the positive correlation between MDM2 expression and Nutlin-3A cytotoxicity in ALL. In fact, a major effect of Nutlin was observed in cells over-expressing MDM2 respect to MDM2-negative ALL cells, probably due to the higher induction of p53, p21, Bax, and PUMA (Gu et al., 2008).

Moreover, Zhu et al. (2008) performed *in vitro* experiments with Nutlin and the inhibitor of antiapoptotic PI3K/AKT pathway that is frequently activated in different cancer cell types. They demonstrated the synergic effect of these drugs in inducing apoptosis in ALL cells.

Recently, we observed the effects of Nutlin-3a in adult B-ALL confirming the activation of p53-mediated pathway in wild-type p53 ALL cells (Trino et al., 2016). Given the clinical significance of BCR-ABL1 mutations in inducing resistance to conventional therapy (Soverini et al., 2016), we analyzed the efficacy of Nutlin-3a in Ph^+^ ALL resistant patients carrying the T315I BCR-ABL1 mutation. Interestingly, we observed that this drug is able to reduce *in vitro* cell viability in this subtype of resistant ALL suggesting its potential therapeutic application in resistant clinical setting of patients (Trino et al., 2016).

Moreover, due to the evidences that ETV6/RUNX1 (E/R), the most common fusion gene in childhood ALL, impaired p53 signaling, Kaindl et al. (2014) investigated the effect of Nutlin in E/R ALL cells. They demonstrated that MDM2 was over-expressed in E/R-positive respect to E/R-negative primary B-cell precursor-ALL samples, showing also that E/R transcription factor binds to the MDM2 P2 promoter and consequently up-regulates MDM2 in a direct and p53-independent manner. Nutlin-3 treatment reactivated p53 function in E/R-expressing leukemic cell lines, leading to cell cycle arrest, enhanced apoptosis, and increased expression of p53 direct targets p21, MDM2, and the pro-apoptotic BAX and PUMA (Kaindl et al., 2014).

Furthermore, Richmond et al. (2015) carried out a preclinical study in a specific subset of infant ALL patients carrying the translocation in the mixed-lineage leukemia (MLL) oncogene, associated with a lower survival rate. They demonstrated that RG7112, the analog of Nutlin-3a, induced regression and prolonged progression delay in a panel of patient-derived infant MLL-ALL xenografts, and p53 upregulation, cell cycle arrest and induction of apoptosis.

Kang et al. (2016) instead tested the efficacy of another inhibitor of MDM2, MK-8242, in *in vitro* and *in vivo* tumor panels and compared this study with their previous evaluation of RG7112 in the same cell line models (Carol et al., 2013). For both agents, they demonstrated that the *in vitro* ALL cell line sensitivity correlated with *TP53* mutation status. Moreover, for *in vivo* experiments, the response of the leukemia xenografts was similar between MK-8242 and RG7112; in particular, xenografts from two MLL-rearranged cell lines achieved or maintained complete responses. Other non-MLL ALL xenografts had partial responses to MK-8242.

Interestingly, emerging literature data reported that MDM2 inhibition played a role not only in apoptosis induction but also in autophagy activation in different hematological malignancies, like multiple myeloma (Gu et al., 2014) and acute myeloid leukemia (AML; Borthakur et al., 2015).

Collectively, these different studies indicated that MDM2 inhibition could be a new promising target therapy in hematological malignancies.

## Use of MDM2 Inhibitors in Combination Setting

Since drug resistance to MDM2 inhibitors or current therapeutic agents can be acquired by tumor cells, pharmacological combination could be a successful strategy to improve the treatment outcome and to reduce the side-effects of the drugs. In this regard, different groups evaluated *in vitro* the combinatory effects between Nutlin-3a and conventional drugs used in ALL therapy. Kaindl et al. (2014) reported that co-exposure of Nutlin-3a and chemotherapeutic drugs (daunorubicin, asparaginase, vincristine) reduced cell viability and potentiated apoptosis in a childhood ALL cell line, with E/R fusion gene.

In our previous study, we evaluated *in vitro* the co-treatment of Nutlin-3a with TKIs in Ph^+^ cell lines. In particular, the combination between Nutlin-3a and Imatinib, Dasatinib or Nilotinib showed significant effect in reducing cell viability of a Ph+ cell line in comparison with the effect of the single TKI treatment (Trino et al., 2016).

Another study by Richmond et al. (2015), showed that combining RG7112 with an induction type regimen (vincristine, dexamethasone, and L-asparaginase) significantly enhanced objective responses and prolonged leukemia regression *in vivo* MLL-ALL xenografts.

On the light of these pre-clinical evidences, literature data underline that targeting the p53-MDM2 axis in combination with established drugs for the management of ALL warrants further investigations.

## MDM2 Inhibitors in Clinical Trials

As previously described, different preclinical studies demonstrated the *in vitro* and *in vivo* effects of MDM2 inhibitors to kill wild-type p53 tumor cells. Therefore, due to their promising anticancer abilities, these drugs are now translated into clinical trials to better assess their biological effects and toxicities in patients. RG7112 was the first MDM2 inhibitor entered clinical evaluation. Recently, a multicenter phase I trial of RG7112 was conducted in patients with hematological malignancies, including ALL (Andreeff et al., 2016). This study confirmed p53 stabilization and transcriptional activation of p53 target genes after MDM2 antagonist treatment, also demonstrating clinical activity in patients with poor prognosis, relapsed, or refractory. To identify the effective biomarkers of response, in this study were evaluated the p53 status by detection of single nucleotide substitution or deletion in exons 2-11 as well as their splice sites. Moreover, mRNA expression, by quantitative real-time PCR, of 24 direct and indirect p53 target genes and MDM2 transcript was also examined. By analyzing patient data the authors did not find any molecular marker predicting response to RG7112. Since this inhibitor was effective in patients with at least 1 wild-type *TP53* allele, *TP53* mutation status alone did not define pharmacological response. Furthermore, baseline MDM2 expression levels were found positively correlated with clinical response, but also this was not sufficient to define MDM2 as a single predictive marker of sensitivity to treatment. The analysis of p53 target genes showed 10, among 24, p53 target genes significantly modulated but only in p53 wild-type samples. Among those, the most induced genes were *CDKN1A*/p21, a crucial p53-mediator of cell-cycle arrest, and *BBC3*/PUMA, an important mediator of p53 dependent apoptosis (Andreeff et al., 2016).

However, from a clinical point of view, RG7112 showed several disadvantages as the gastrointestinal intolerance due to a high dose required for drug efficacy and variability of exposure at the maximum tolerate dose. To overcome these limitations, recently a new potent MDM2 inhibitor RG7388, also known as Idasanutlin, has been discovered (Ding et al., 2013) and actually entered in a phase 1/1b study in relapsed/refractory AML. Recent data about this trial revealed that MDM2 protein expression levels in leukemic blasts and stem cells were associated with Idasanutlin-induced complete remission in AML patients (Reis et al., 2016). Moreover, the same trial evaluated Idasanutlin as monotherapy or in combination with cytarabine in relapsed/refractory AML patients (Reis et al., 2016). No current data are available on ALL.

## Conclusion

**FIGURE 1:**
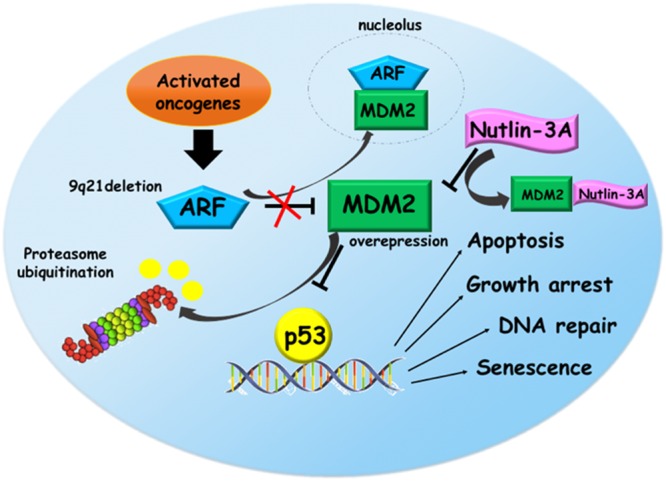
**Reactivation of p53 pathway via Nutlin-3a in acute lymphoblastic leukemia (ALL).** In response to oncogenic activation, ARF protein interacts with MDM2 sequestering it into the nucleolus. This binding prevents the proteasomal degradation of p53 that activates its target genes promoting several functions like apoptosis, growth arrest, DNA repair, and senescence. In ALL, *9p21* locus deletion and MDM2 overexpression eliminate the tumor surveillance mechanism based on ARF-MDM2 interaction leading to the p53 degradation. Nutlin-3a, a small molecule targeting MDM2, restores p53 pathway, suggesting a promising therapeutic option for ALL.

P53 pathway is often altered in ALL, in particular due to the overexpression of *MDM2* and deletion of *CDKN2A*, the two main regulator of p53. Thus, targeting of MDM2-p53 axis could represent an attractive cancer therapeutic strategy in ALL. Nodaway, potent and selective MDM2 inhibitor drugs are available, such as Nutlins (**Figure 1**). These small molecules not only showed a preclinical evidence to restore p53 pathway, but also had a pharmaceutical properties and entered into clinical trials.

Clinical testing of Nutlin-3a and new agents activating p53 tumor suppressor functions may provide proof of concept for their therapeutic approaches in ALL.

## Author Contributions

ST and LDL revised the literature available on this topic and wrote the paper; IL and AC contributed in the scientific writing of the manuscript; LDV, GM, and PM revised the manuscript. All authors approved the paper for publication.

## Conflict of Interest Statement

The authors declare that the research was conducted in the absence of any commercial or financial relationships that could be construed as a potential conflict of interest.

The reviewer AR and handling Editor declared their shared affiliation, and the handling Editor states that the process nevertheless met the standards of a fair and objective review.
